# Precision non-invasive transcranial electromagnetic stimulation: a thematic review of optimizing spatial, temporal, and dose dimensions

**DOI:** 10.1007/s00406-026-02256-4

**Published:** 2026-04-29

**Authors:** Ruixuan Lin, Weijia He, Usman Jawed Shaikh, Lukas Lorentz, Roy Rongyue Zeng, Patrick W. H. Kwong, Tiev Miller, Pablo Cruz Gonzalez, Ananda Sidarta, Ferdinand Binkofski, David M. A. Mehler, Jack Jiaqi Zhang

**Affiliations:** 1 Department of Rehabilitation Sciences, The Hong Kong Polytechnic University, Hung Hom, Hong Kong S.A.R., China; 2 Department of Rehabilitation Medicine, School of Medicine, the First Affiliated Hospital of Xiamen University, Xiamen University, Xiamen, China; 3 Division of Clinical Cognitive Sciences, Department of Neurology, University Hospital RWTH Aachen, Aachen, Germany; 4 Rehabilitation Research Institute of Singapore, Nanyang Technological University, Singapore, Singapore; 5 Department of Psychiatry, Psychotherapy and Psychosomatics, University Hospital RWTH Aachen, Aachen, Germany; 6 Institute for Translational Psychiatry, University of Münster, Münster, Germany

**Keywords:** Brain stimulation, Neuroplasticity, Neuromodulation, Target engagement, Dose optimization, Rehabilitation

## Abstract

Transcranial magnetic stimulation (TMS) and transcranial electrical stimulation (TES) have been extensively used in experimental and clinical research in psychiatry, neurology, and rehabilitation. Several advanced approaches for delivering transcranial electromagnetic stimulation have been proposed to maximize the temporal and spatial precision of conventional stimulation protocols or to optimize stimulation parameters for enhanced efficacy during neuromodulation. Here, we thematically reviewed several advanced neuromodulatory approaches: (1) using prior or real-time brain states to guide the delivery of stimulation through priming protocols or brain-state-dependent stimulation interfaces (temporal precision); (2) facilitating the stimulation target engagement, either from a single brain region to an interregional brain connection using cortico-cortical paired associative stimulation or dual-site in/anti-phase transcranial alternating current stimulation, or from a superficial brain target to a deep brain region using temporal interference stimulation (spatial precision); and (3) strengthening or accelerating the plasticity modulation outcomes using high-dose protocols (dose optimization). While results from these efforts are promising, challenges exist in terms of the optimal timing of brain states and stimulation parameters, treatment durability, as well as the complexity in aggregating different advanced approaches. Future research should focus on exploring the underlying mechanisms, optimizing parameters, and assessing the efficacy in well-designed proof-of-concept experiments and clinical trials.

## Introduction

Transcranial magnetic stimulation (TMS) and transcranial electrical stimulation (TES) have emerged as mainstream non-invasive brain stimulation (NIBS) techniques extensively employed in experimental and clinical research across neurology, psychiatry, and rehabilitation [1–3], with certain protocols receiving regulatory approval for clinical treatment—primarily for psychiatric disorders—in specific countries and regions [4]. The repertoire of NIBS protocols has expanded substantially, with several advanced neuromodulatory strategies and approaches developed to enhance the efficacy and specificity of neuroplasticity induction. These advancements aim not only to improve therapeutic outcomes but also to facilitate broader clinical translation across diverse neurological and psychiatric conditions. As an extension of a previous review [5], the present work synthesizes the current literature on advanced transcranial electromagnetic stimulation protocols designed to optimize temporal, spatial, and dose-related dimensions. This review outlines their definitions and presumed or established mechanisms of action, and evaluates existing evidence derived from representative proof-of-concept human studies, as well as pilot and full-scale clinical trials. A concluding discussion is provided accordingly.

## Advanced protocols

The therapeutic NIBS interventions employed in prior experimental and clinical research include repetitive TMS (rTMS) and its specialized forms—theta burst stimulation (TBS) and quadripulse stimulation (QPS)—along with transcranial alternating current stimulation (tACS), transcranial direct current stimulation (tDCS), and transcranial random noise stimulation [6–9]. Advanced protocols have subsequently emerged to further enhance spatial and temporal precision and optimize stimulation parameters, such as dose, thereby demonstrating considerable potential to produce more robust neuromodulatory effects.

**Fig. 1:**
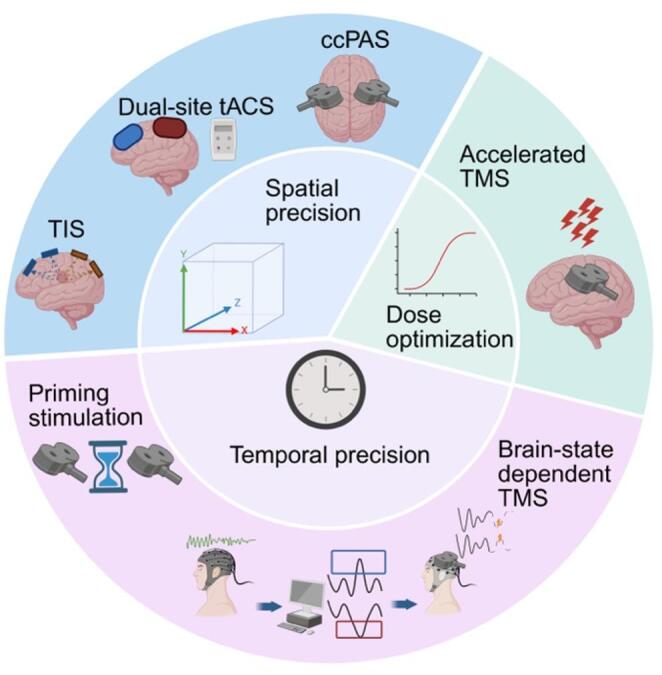
A conceptual framework for optimizing neuromodulation. Enhancing interventional efficacy could be achieved by targeting three core domains: spatial precision (via techniques such as ccPAS, dual-site tACS, and TIS), dose optimization (e.g., accelerated TMS), and temporal precision (e.g., priming stimulation and brain-state-dependent TMS). The figure was created with BioRender.com (agree number: XB29FB7MLI). *ccPAS* corticocortical paired associative stimulation; *tACS* transcranial alternating current stimulation; *TMS* transcranial magnetic stimulation; *TIS* temporal interference stimulation

To provide a structured framework for these innovations, we categorize advanced neuromodulatory protocols into three strategic dimensions based on their primary mechanisms of enhancement (Fig. 1).

- First, we examine advancements in spatial precision designed to target deep structures or modulate specific interregional connectivity. These approaches include corticocortical paired associative stimulation (ccPAS) [10] and dual-site phase-dependent tACS [11], which appear to extend reliable target engagement from a single region to interregional connections. Additionally, temporal interference stimulation (TIS) appears to enable selective modulation of deep neuronal populations while minimizing effects on superficial cortical areas [12].
- Second, we discuss methods that enhance temporal precision by synchronizing stimulation with the brain’s ongoing or prior physiological activity. These include metaplasticity-induced priming stimulation, in which sequential non-identical protocols are employed; the initial session modulates the long-term potentiation/long-term depression (LTP/LTD)-like plasticity threshold to enhance responsiveness to the subsequent protocol via metaplasticity [13]. This dimension also encompasses the use of brain-state–dependent neuromodulatory interfaces that deliver stimulation based on real-time neural activity to optimize intervention timing [14].
- Finally, we address parameter optimization in advanced NIBS protocols. Although the parameters available for exploration are theoretically infinite, dose optimization has been the most extensively studied and is closest to clinical translation. Therefore, this review focuses specifically on how increased dosage may facilitate cumulative plasticity. This approach aims to enhance both neurophysiological and clinical outcomes by increasing pulse delivery within conventional protocols, based on a presumed dose-dependent relationship between stimulation exposure and modulatory effects [15].

### Enhancing spatial precision: deep targeting and connectivity modulation

**Fig. 2:**
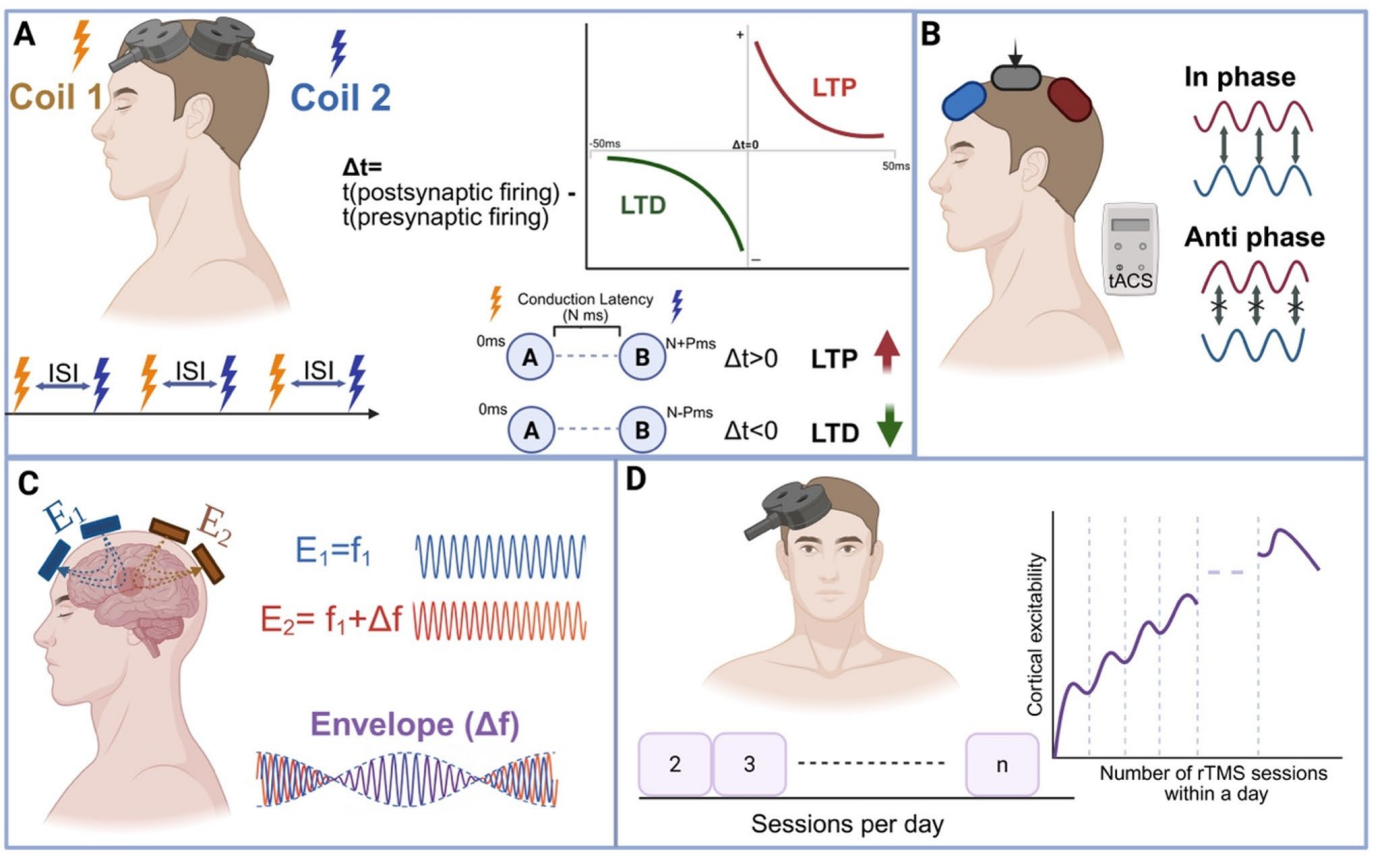
Spatial precision **A** Corticocortical paired associative stimulation, **B** Dual-site transcranial alternating current stimulation, **C** Temporal interference stimulation, **D** Accelerated transcranial magnetic stimulation. The figure was created with BioRender.com (agree number: KU29FB7M4M). *ISI* Interstimulus interval; *LTP* long-term potentiation; *LTD* long-term depression; *tACS* transcranial alternating current stimulation; *rTMS* repetitive transcranial magnetic stimulation

Spatial precision in NIBS has benefited from the integration of neuronavigation. However, two major spatial challenges remain: how to noninvasively and focally modulate deep brain structures, and how to selectively influence specific interregional connectivity (Fig. 2).

#### Targeting deep brain structures

TIS, introduced by Grossman et al. [12], is a non-invasive neuromodulatory technique that enhances spatial precision by selectively targeting deep brain regions. TIS involves the application of two high-frequency sinusoidal currents with slightly different frequencies—for example, 2 kHz and 2.01 kHz. Owing to this frequency difference, these waves interfere to generate a low-frequency amplitude-modulated envelope (e.g., 10 Hz) (Fig. 2C). As neurons cannot directly follow high carrier frequencies, superficial tissues remain largely unaffected, whereas the envelope modulates neural activity within deeper structures [16].

In recent years, TIS has attracted considerable research interest and has been increasingly applied in human studies. Evidence suggests that TIS effectively modulates neuronal activity in both superficial and deep brain regions (e.g., the hippocampus) [12]. Application of 2 mA TIS for 20 min over the primary motor cortex (M1) has been shown to produce effects comparable to those of tDCS and to enhance functional connectivity between M1 and the secondary motor cortex in healthy individuals [17]. In another experiment involving stimulation of the right parietal lobe, TIS improved working memory performance in healthy individuals and demonstrated effects similar to those of tACS [18]. Both studies indicate that TIS can achieve neuromodulatory effects comparable to conventional TES protocols when targeting superficial cortical regions. Applications of TIS in non-invasive deep brain stimulation have also yielded promising findings. A study by Wessel et al. demonstrated that theta-burst–patterned TIS targeting the human striatum significantly enhanced striatal and motor network activity, thereby improving motor skill acquisition in a sequential finger-tapping task, particularly in older adults. Electrophysiological and blood-oxygen-level-dependent (BOLD) signal analyses have confirmed that TIS effects are largely confined to the striatum and its associated networks, with minimal impact on overlying cortical regions [19]. In another experiment targeting the hippocampus with TIS, computational modeling and human cadaver studies demonstrated that TIS can precisely target deep brain structures, yielding a 30–60% higher envelope modulation amplitude in the hippocampus than in cortical regions, with adjustable focalization along the hippocampal axis via electrode configuration. Functional magnetic resonance imaging (fMRI) combined with a face–name association task revealed that TIS significantly reduced hippocampal BOLD signals during memory encoding, indicating selective modulation of neural activity. Behavioral data further suggest that TIS may improve episodic memory accuracy [20]. To date, TIS has been applied in clinical populations, with a few pilot studies indicating potential utility in movement disorders (e.g., Parkinson’s disease and essential tremor) [21–24] and bipolar disorders [25]. In summary, current TIS research consists primarily of proof-of-concept studies in healthy individuals and early pilot clinical trials. However, its clinical efficacy requires further investigation through robust randomized controlled trials (RCTs).

#### Targeting interregional connectivity

Many neurological and psychiatric disorders are associated with disrupted neural connectivity and network-level dysfunction rather than abnormalities confined to a single brain region [26, 27]. Therefore, reliable neuromodulation of interregional brain connectivity is likely to maximize the therapeutic efficacy of NIBS in the management of specific clinical symptoms.

In an early study, bifocal high-frequency rTMS trains were simultaneously applied to the M1 and primary visual cortex (V1) in healthy adults, resulting in a facilitatory effect on M1–V1 interregional coherence as measured by electroencephalography (EEG) [28]. In addition to the simultaneous application of high-frequency trains to two targets, ccPAS is another technique that involves the repetitive, sequential delivery of paired-pulse stimulation to two (or more) interconnected brain regions [29]. Building on these foundations but incorporating precisely timed delays (Fig. 2A), ccPAS is based on spike-timing-dependent plasticity (STDP) [30], which posits that LTP occurs when presynaptic neurons repeatedly activate before postsynaptic neurons, whereas LTD arises when presynaptic activation repeatedly follows postsynaptic activation [31]. Human studies employing ccPAS have demonstrated STDP-like plasticity between structurally connected cortical regions. In ccPAS, connectivity potentiation (LTP-like effects) is induced when the interstimulus interval (ISI) matches the physiological conduction delay of a structurally connected circuit and the stimulation sequence follows the natural direction of neuronal signaling. Conversely, a mismatched ISI or reversed stimulation order may result in synaptic depression (LTD-like effects) (Fig. 2A). According to the literature, ccPAS appears to modulate connections between brain regions such as the bilateral M1; ventral premotor cortex (PMv)–M1; supplementary motor area (SMA)–M1; posterior parietal cortex (PPC)–M1; visual pathways; and the frontoparietal connectivity, some of which have been shown to influence cortical excitability and interregional excitation–inhibition balance [32]. ccPAS has also been applied in clinical trials. One study involving patients with generalized anxiety disorder used ccPAS to target the right dorsolateral prefrontal cortex (DLPFC) and right PPC [33]. Each site received 15 trains of 100 pulses at intertrain intervals of 30 seconds. The intervention resulted in significant reductions in clinical scores for anxiety, depression, and insomnia, demonstrating superior efficacy compared with single-site stimulation. In another trial applying ccPAS over the SMA–M1 pathway (0.2 Hz, 180 pulses) in poststroke patients, the intervention demonstrated superior efficacy compared with sham stimulation in facilitating upper limb motor recovery and strengthening connectivity within the ipsilesional sensorimotor system [34].

A key barrier to the use of dual-coil TMS in connectivity modulation research has been the physical size of conventional coils, which limited the simultaneous stimulation of two nearby brain regions. Recent technological advancements have substantially facilitated the investigation of ccPAS. In particular, the development of specialized small-sized TMS coils and dual-coil navigation systems has enabled the precise and simultaneous stimulation of closely spaced cortical targets, leading to a growing body of ccPAS studies.

Compared with TMS, the TES family offers an additional approach for modulating brain connectivity. Dual-site tACS has been shown to modulate connectivity between brain regions in human participants by placing two active electrodes over distinct cortical target areas and a reference electrode over an electrically neutral site (Fig. 2B). In this configuration, in-phase stimulation (0° phase difference between the two tACS currents) is thought to facilitate neuronal synchronization, whereas anti-phase stimulation (180° phase difference) is thought to desynchronize neuronal communication [35]. Dual-site tACS applied at different frequencies has been associated with various cognitive and motor functions; for example, beta-band stimulation can be linked to motor control [36–38], while theta-band stimulation is often associated with cognitive processes, such as memory [39, 40]. Human experiments have demonstrated that dual-site tACS is effective in modulating both intrahemispheric [39, 41] and interhemispheric interactions in healthy individuals [37, 38, 42]. Dual-site tACS has also been applied in clinical populations, including individuals with poststroke aphasia [43], mild cognitive impairment [40] and drug addiction [44].

In conclusion, advanced stimulation protocols such as ccPAS and dual-site tACS are likely to modulate brain connectivity and thereby improve clinical conditions associated with network-level impairment. This approach is currently supported by human proof-of-concept studies and a limited number of early-stage clinical trials. However, the protocols applied in different studies are diverse and therefore require external validation.

### Enhancing temporal precision: the impact of brain state

**Fig. 3:**
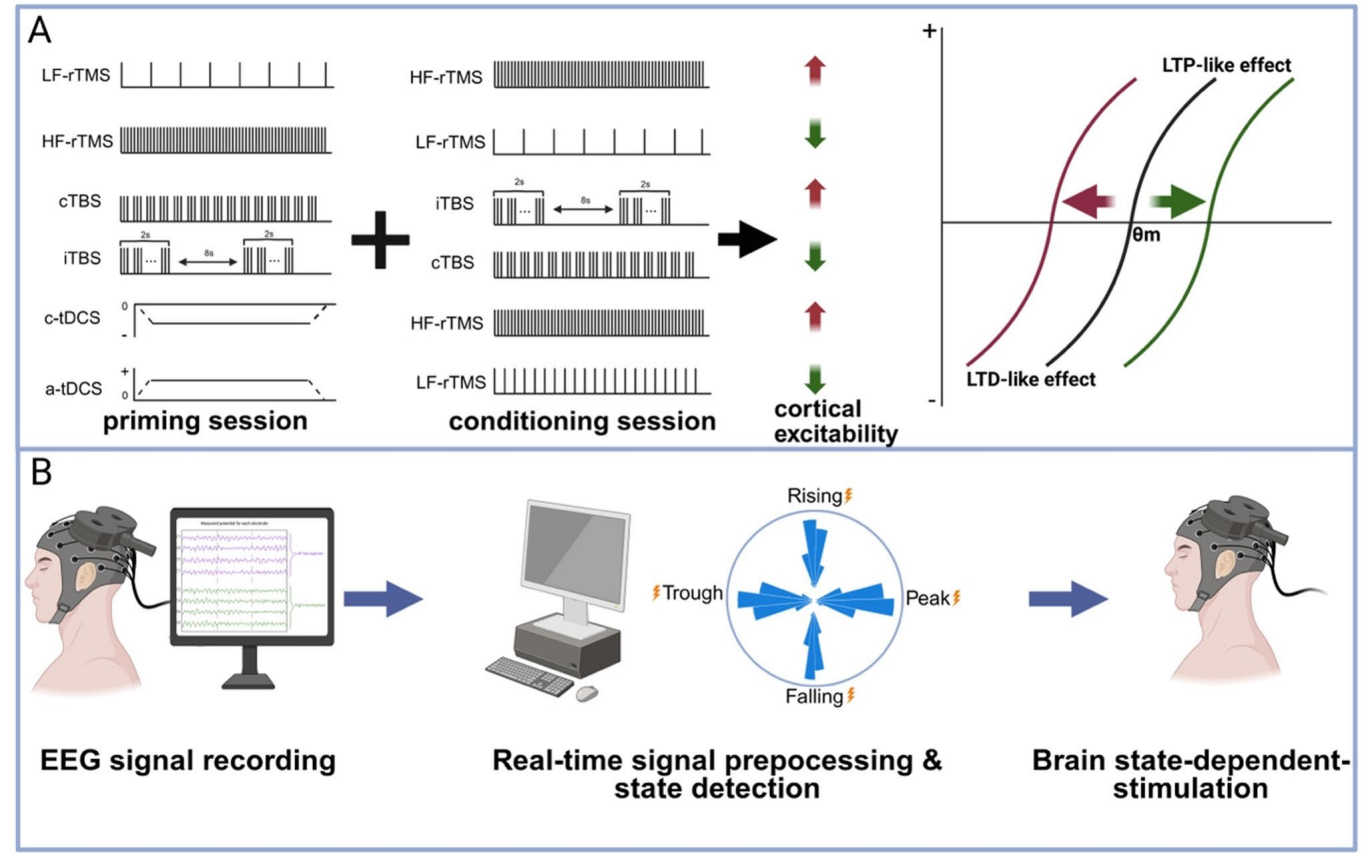
Temporal precision. **A** The paradigm and mechanism of metaplasticity-induced priming stimulation, **B** EEG-TMS based brain-state-dependent stimulation. The figure was created with BioRender.com (agree number: ZB29FB7LQ2)

Conventional NIBS protocols typically deliver stimulation without consideration of brain state. However, neuronal activity is inherently dynamic, and this ongoing activity fundamentally shapes neuronal responses to external stimuli; therefore, the efficacy of neuromodulation is critically dependent on the timing of stimulation relative to the brain’s intrinsic activity [14]. Evidence that brain state influences responses to neuromodulation has motivated the development of brain-state–informed stimulation protocols. By harnessing prior or ongoing neural activity, these advanced methods aim to enhance the temporal precision of NIBS (Fig. 3).

#### Utilizing prior brain states: metaplasticity-induced priming stimulation

Priming stimulation involves two non-identical TMS or tDCS protocols, with the first serving as a priming intervention followed by conditioning stimulation (Fig. 3A). Its underlying mechanism is the induction of metaplasticity. Metaplasticity refers to the capacity of synapses to alter their responses based on previous synaptic activity [45, 46]. Prior synaptic activity influences the brain’s response to NIBS, such that lower levels of prior activity may lead to stronger LTP effects, whereas higher levels of prior activity favor stronger LTD induction. In essence, metaplasticity highlights how prior synaptic activity shapes synaptic responses to subsequent stimuli and therefore should be considered when designing priming stimulation protocols [45, 46].

The initial priming protocol employed rTMS and consisted of a session of high-frequency stimulation (6 Hz), followed by a session of low-frequency stimulation (1 Hz). This priming protocol was previously shown to produce greater inhibition of motor-evoked potentials (MEPs) in healthy adults than low-frequency rTMS alone (non-priming control) [13]. Subsequently, numerous proof-of-concept studies in healthy individuals using various stimulation protocols—including rTMS, TBS, QPS, and anodal or cathodal tDCS—demonstrated metaplasticity in the M1 as assessed by MEPs [47–49]. Although metaplasticity-like effects have primarily been demonstrated in proof-of-concept human studies targeting M1 using MEPs, they have also been observed in other brain regions, such as the DLPFC. For example, an fMRI study applied cathodal tDCS to prime intermittent theta burst stimulation (iTBS) targeting the DLPFC and demonstrated enhanced functional connectivity between the DLPFC and striatum compared with sham tDCS–primed iTBS in healthy adults [50]. Therefore, metaplasticity-induced priming stimulation—whether via “inhibition before excitation” or “excitation before inhibition”—appears to enhance modulatory outcomes.

Priming stimulation has been explored in several clinical trials involving populations with various disorders. For instance, an RCT demonstrated that continuous TBS (cTBS)-primed iTBS enhances treatment gains from robot-assisted upper limb motor therapy in post-stroke patients to a greater extent than non-primed iTBS or sham stimulation [51]. The study found that both primed and non-primed stimulation improved upper limb motor recovery in post-stroke patients compared with sham stimulation, with higher-functioning patients deriving greater benefit from cTBS-primed iTBS [51]. A recently published RCT protocol based on a similar concept proposed the application of cathodal tDCS prior to anodal tDCS in post-stroke patients. The authors hypothesized that inhibitory priming using cathodal tDCS may amplify the excitatory effects of anodal tDCS [52], thereby leading to more favorable upper limb recovery outcomes. Moreover, priming rTMS (using 6 Hz rTMS as priming and 1 Hz rTMS as treatment) has also shown promising results in the treatment of major depressive disorder (MDD). A clinical study demonstrated that a prior excitatory priming session enhanced the effect of 1 Hz rTMS in improving depressive symptoms in patients with MDD, presumably through metaplasticity [53].

Supported by small-to-moderate sample-size clinical trials, metaplasticity-induced priming represents a promising approach to enhance the therapeutic effects of subsequent M1- or DLPFC-based stimulation in neurological and psychiatric disorders. Nevertheless, further research is required to optimize the parameters of metaplasticity-induced protocols, such as stimulation intensity and interstimulus interval, in order to minimize variability in treatment response.

#### Synchronizing with real-time brain states: brain-state–dependent stimulation

In addition to priming stimulation, which utilizes prior neural activity, real-time brain-activity–guided stimulation offers promise for more temporally precise neuromodulation [54]. EEG-triggered TMS is a brain-state–dependent technique that synchronizes magnetic pulse delivery with specific physiological brain states to induce LTP- or LTD-like cortical plasticity. A representative example in this field is EEG sensorimotor mu (µ)-rhythm–triggered TMS [55] (Fig. 3B). This study demonstrated that when a single TMS pulse was applied at the trough of the µ-rhythm, it elicited larger MEP amplitudes compared with pulses delivered at the peak or at random phases. Furthermore, repetitive magnetic stimulation delivered at the trough of the µ-rhythm—but not at other oscillatory phases—enabled EEG-triggered trains of magnetic pulses to induce LTP-like effects in healthy individuals [55]. These observations are interpreted such that negative deflections in spontaneous EEG reflect superficial postsynaptic excitatory potentials, whereas positive deflections represent superficial postsynaptic inhibitory potentials [56]. Moreover, a clinical RCT implemented brain-state–dependent rTMS in post-stroke patients by triggering TMS bursts at the trough of the ipsilesional sensorimotor µ-rhythm, guided by real-time EEG signals. The results demonstrated that the brain-state–dependent rTMS group achieved therapeutic effects comparable to those of the established 1 Hz rTMS protocol targeting the contralesional M1 in improving motor function and reducing spasticity during post-stroke rehabilitation [57]. EEG-triggered TMS has also been successfully applied to other oscillatory rhythms and brain regions, such as theta-oscillation–guided stimulation of the left dorsomedial prefrontal cortex (DMPFC) [58]. The authors reported differential effects of positive- and negative-phase theta-oscillation–guided repetitive triplet TMS trains on theta oscillatory power, with negative-phase–guided stimulation improving working memory performance in healthy adults [58]. Furthermore, a recently published RCT protocol proposed the use of DMPFC negative-phase theta-oscillation–guided repetitive burst trains versus standard iTBS for depression treatment, highlighting the emerging clinical application of brain-state–dependent stimulation in psychiatry [59].

Brain-state–dependent stimulation has also been integrated with ccPAS for dual-site targeting. One study demonstrated that, in seven healthy individuals, brain-state–dependent ccPAS targeting the frontal and parietal regions significantly improved working memory accuracy, whereas open-loop ccPAS did not. This connectivity-based protocol used the phase of theta oscillations as a brain-state marker, triggering magnetic pulses during the positive peak of the theta rhythm based on real-time EEG analysis [60]. Another study applied brain-state–dependent ccPAS to modulate interhemispheric inhibition (IHI) between the bilateral motor cortices [61]; however, different phase-controlled ccPAS conditions did not differ from open-loop ccPAS in modulating IHI. These findings highlight the potential of integrating spatial (ccPAS) and temporal precision (EEG-triggered) protocols in NIBS.

In summary, EEG-triggered TMS represents the predominant approach currently available for brain-state–dependent stimulation, with potentially important clinical implications for targeted neuromodulatory therapy. However, the existing literature consists predominantly of proof-of-concept studies in healthy individuals, with a very limited number of pilot clinical investigations.

### Parameter optimization

The parameters available for exploration are theoretically infinite, including stimulation intensity, pulse width, and waveform shape. Among these, optimization of stimulation dose has been the most extensively studied and is closest to clinical translation.

#### Intensifying stimulation dosage and shortening treatment duration: accelerated protocols

Therapeutic NIBS protocols may accelerate behavioral outcomes or strengthen plasticity modulation by increasing the stimulation dose and/or the number of sessions [62]. This proposition is supported by several meta-regression studies, in which a greater number of stimulation pulses was associated with larger effect sizes across various clinical outcomes [63, 64]. Accelerated stimulation involves either increasing the number of pulses per session, delivering multiple sessions per day, or both, thereby shortening treatment duration while potentially achieving comparable or more robust therapeutic effects (Fig. 2D).

Both conventional rTMS and TBS protocols can be implemented using accelerated paradigms. The first study to directly compare accelerated and conventional rTMS in the treatment of depression was published in 2018 [65]. This study delivered three rTMS sessions per day over three days, administering a total of 10,500 pulses per day across the three sessions (63,000 pulses in total). The findings indicated that accelerated rTMS was as effective as conventional 20-session rTMS protocols, with both delivering 3,150 pulses per day and 63,000 pulses in total [65]. TBS is more frequently employed in accelerated paradigms because its stimulation duration is substantially shorter than that of rTMS, thereby reducing overall treatment time. The effects of 600, 1,200, and 1,800 pulses of iTBS have been compared in terms of TMS-evoked potentials over the DLPFC in healthy adults [66] and clinical outcomes in patients with depression [67]. However, neither study identified significant differences among iTBS protocols using varying pulse numbers per session [66, 67]. Accelerated TBS studies that consolidate multiple stimulation sessions into a single day highlight its potential as a clinical intervention. The Stanford Accelerated Intelligent Neuromodulation Therapy (SAINT) protocol is a well-established accelerated iTBS approach for treating depression. It administers 10 sessions of 1,800-pulse iTBS per day for five consecutive days and targets the DLPFC region showing the strongest anticorrelation with the subgenual anterior cingulate cortex, as identified using resting-state fMRI. The 50-minute interval between stimulation sessions was presumed sufficient to facilitate spaced learning and prevent homeostatic plasticity when identical protocols (e.g., iTBS) were applied [68]. Studies have demonstrated that the SAINT protocol achieves high remission rates in depression [69, 70]. Moreover, a recent study employing a lower-dose accelerated TBS protocol, consisting of three iTBS sessions per day, also demonstrated superiority in depression treatment. These findings suggest that dose-dependent plasticity induction may occur even at relatively lower doses, and the three-session protocol may be more practical than the SAINT protocol in certain clinical contexts [71]. In addition to depression, accelerated iTBS may exert therapeutic effects on substance-use craving [72, 73] and cognitive impairment [74, 75]. In summary, accelerated protocols—most notably the SAINT protocol—have progressed toward clinical translation, and RCTs evaluating their efficacy in MDD are now available.

## Discussion

This review provides a comprehensive synthesis of several advanced NIBS approaches: (1) leveraging prior or real-time brain states to guide stimulation delivery through priming protocols or brain-state–dependent interfaces (temporal precision); (2) enhancing target engagement either by extending stimulation from a single brain region to interregional connections using ccPAS or dual-site phase-dependent tACS, or by shifting from superficial cortical targets to deep brain structures using TIS (spatial precision); and (3) strengthening or accelerating plasticity-modulation outcomes using intensive protocols (parameter optimization).

### Spatial precision

Recent technological advances have redefined spatial targeting by shifting the emphasis from focal cortical stimulation toward deep-structure engagement and network-level modulation. TIS targets deeper brain structures by generating interference-based electric fields that are concentrated at depth. In contrast, ccPAS emphasizes network connectivity rather than modulation of individual regions. ccPAS has shown considerable potential for influencing motor behavior, visual discrimination, cognitive function, and functional connectivity [32, 76], although most studies have been conducted in healthy individuals. Its therapeutic benefits in clinical populations remain to be established. For TIS, several unresolved issues warrant caution. First, a trade-off exists between depth and focality: achieving sufficient field strength at depth often compromises spatial resolution, potentially producing an interference zone larger than intended. Second, interindividual anatomical variability poses a challenge because electric-field distributions are highly sensitive to head geometry and tissue conductivity; therefore, individualized electric-field modeling is likely required to ensure reliable target engagement [16]. Third, stimulation intensity remains a constraint. Because high-frequency currents are substantially shunted by superficial tissues, higher amplitudes may be required to reach deep targets, and current attenuation can occur as kilohertz-frequency signals propagate through the head and brain [77]. This requirement may raise safety and tolerability concerns (e.g., cutaneous sensations). Therefore, progression from proof-of-concept studies to reliable clinical application requires rigorous attention to these biophysical constraints and sources of methodological heterogeneity.

### Temporal precision

With respect to temporal precision, advanced protocols have increasingly focused on delivering stimulation at time points when the brain exhibits optimal responsiveness to neuromodulatory input. These approaches place greater emphasis on the brain state during stimulation. Priming stimulation, an open-loop strategy, aims to establish a suitable brain state prior to conditioning stimulation in order to induce stronger modulatory effects. Although several clinical trials have reported positive outcomes with priming stimulation, MEP studies in healthy individuals have yielded mixed results regarding its capacity to enhance subsequent stimulation effects, most likely due to variability in time intervals and stimulation intensity [47]. Therefore, parameter optimization remains an essential objective for future research on priming stimulation [78].

In contrast, brain-state–dependent protocols deliver stimulation according to specific neural states via brain–stimulator interfaces in an effort to enhance therapeutic efficacy. However, identifying optimal time intervals, stimulation intensities, and the appropriate combination of protocols for both state modulation and conditioning stimulation remains an area requiring further investigation. Within these approaches, selecting appropriate EEG indicators for stimulation timing and predicting the most effective intervention windows necessitates extensive experimental and clinical validation. A key technical challenge lies in improving hardware and software systems to facilitate clinical translation. Furthermore, brain state is largely determined by ongoing cognitive, behavioral, and emotional processes [79], which significantly influence the brain’s response to TMS. Currently, brain-state–dependent stimulation—such as EEG-triggered TMS—primarily relies on resting-state neurophysiological markers, including stimulation triggered at specific oscillatory phases or power levels. Integrating real-time EEG triggers with concurrent behavioral tasks may synergistically enhance plasticity, although such integration would inevitably increase experimental complexity. In addition, currently available EEG-triggered TMS interfaces do not yet constitute true closed-loop systems, as the outcome of each stimulation is not used to adapt subsequent pulses [80].

### Dose optimization

Accelerated stimulation has demonstrated considerable promise in recent clinical trials. However, caution remains warranted, as the nature of accelerated protocols may amplify placebo effects associated with rTMS. A recent meta-analytic review reported that device-based neuromodulation produces significantly larger placebo responses than pharmacotherapy, with effects scaling according to procedural complexity; among these, rTMS is associated with a substantial placebo response in the treatment of depression [81]. Distinguishing true physiological effects from these non-specific placebo responses in accelerated protocols remains essential and may be facilitated by studies employing neurophysiological [82] or neuroimaging techniques [83]. Additionally, studies incorporating long-term follow-up data are warranted to evaluate the cost-effectiveness of this treatment paradigm. In summary, accelerated stimulation protocols involving multiple high-dose sessions per day show promise in expediting therapeutic outcomes in individuals with neuropsychiatric conditions. However, given the potential influence of placebo responses and the current lack of long-term follow-up data, both the true therapeutic efficacy and durability of treatment effects require further investigation.

### Further advances

Recent studies have explored the integration of both spatial and temporal dimensions in novel NIBS techniques to optimize therapeutic outcomes. For example, combining brain-state–dependent systems with connectivity-based stimulation (e.g., ccPAS) enables real-time, brain-state–dependent modulation of interregional connections [60]. Similarly, the integration of TIS with TBS has demonstrated robust modulatory effects within deep brain regions [19]. However, integrating these approaches remains biologically complex. For instance, a recent study by Ermolova et al. [61] reported that adding EEG phase-controlled ccPAS did not yield a differential effect compared to open-loop cPAS. The authors discussed that this null finding likely occurred because the stimulation target was determined by MEPs, which are primarily mediated by neurons in cortical layer V, whereas IHI is primarily mediated by neurons in layers II/III. As a result, the stimulation preferentially targeted different neural populations, leading to distinct physiological outcomes. Therefore, the successful integration of multiple advanced stimulation methods requires a deeper understanding of the mechanisms underlying each individual protocol, as well as careful experimental design when combining different methods or applying them in different scenarios. Additionally, future brain-state–dependent research should explore additional signal modalities beyond spontaneous EEG, such as functional near-infrared spectroscopy (fNIRS) and fMRI. Both block- and event-related fMRI and fNIRS can capture task-specific brain states, providing valuable neurophysiological information to guide TMS delivery. Notably, fMRI- and fNIRS-compatible TMS systems are currently available for concurrent measurement [84–86], although existing implementations do not yet support brain-state–dependent stimulation paradigms. Consistent and standardized reporting across stimulation protocols is also essential.

### Limitations

While this review synthesizes advanced NIBS approaches, study selection emphasized key developments rather than adhering to strict inclusion criteria. This approach may introduce bias or result in the omission of relevant studies.

## Conclusion

This review provides a comprehensive overview of advanced NIBS approaches aimed at enhancing stimulation timing through brain-state–guided protocols (EEG-triggered TMS interfaces and priming stimulation), spatial targeting innovations (ccPAS, dual-site tACS, and TIS), and parameter optimization (accelerated protocols). Future research should prioritize mechanistic insights and biomarker-driven stimulation approaches. Additional clinical trials are warranted where proof-of-concept evidence has been well established.
